# Berotralstat effectiveness and safety in patients with hereditary angioedema with normal C1 inhibitor

**DOI:** 10.1016/j.jacig.2025.100575

**Published:** 2025-09-30

**Authors:** Matthew S. Buckland, Isabelle Boccon-Gibod, Claire De Moreuil, Sébastien Sanges

**Affiliations:** a Immunology Department, Royal London Hospital, Barts Health NHS Trust, London, United Kingdom; b Internal Medicine Department, French National Reference Center for Angioedema Centre de Référence des Angiœdèmes à Kinines (CREAK), ACARE, Grenoble Alpes University Hospital, Grenoble, France; c Département de Médecine Interne, Centre Hospitalier Régional Universitaire de Brest, Brest, France; d Département de Médecine Interne et Immunologie Clinique, Centre Hospitalier Universitaire Lille, Lille, France; e CREAK, Lille, France; f University of Lille, Institute for Translational Research in Inflammation (INFINITE)-U1286, Lille, France; g Institut National de la Santé et de la Recherche Médicale, Lille, France

**Keywords:** Angioedema, berotralstat, bradykinin, C1 inhibitor, hereditary, kallikrein

## Abstract

**Background:**

Hereditary angioedema (HAE) with normal C1 inhibitor (nC1-INH) is the least common endotype of HAE, a rare disorder with localized, intermittent attacks of soft tissue swelling. Prophylactic treatments are available across HAE endotypes, including berotralstat, a once-daily oral inhibitor of plasma kallikrein.

**Objective:**

This European multicenter case series aimed to report treatment-related outcomes with berotralstat in patients with HAE-nC1-INH.

**Methods:**

A retrospective case series analysis including observational data from patients in United Kingdom and French centers was performed. Patients were included if they had a genetic assay demonstrating a known HAE-nC1-INH–associated variant; or had a family history of angioedema and normal C1-INH level and function, no response to antihistamine treatment, met predefined diagnostic criteria for HAE-nC1-INH, and had been prescribed berotralstat 150 mg daily for at least 6 months. Data were collected from the patients’ records using a standardized form.

**Results:**

Four female and 2 male patients with HAE-nC1-INH were included from 4 centers in France and the United Kingdom. Duration of berotralstat treatment at analysis ranged from 6 to 23 months. Five patients showed a response to berotralstat, observed as a 29-100% reduction in attack rates. Three patients experienced a reduction in attack severity from moderate to minor or mild. One patient reported adverse events during berotralstat initiation: nausea and diarrhea.

**Conclusion:**

Long-term prophylactic berotralstat was effective in reducing HAE attacks for 5 of 6 patients with HAE-nC1-INH. No significant safety signals were noted.

## Graphical abstract

**Figure:**
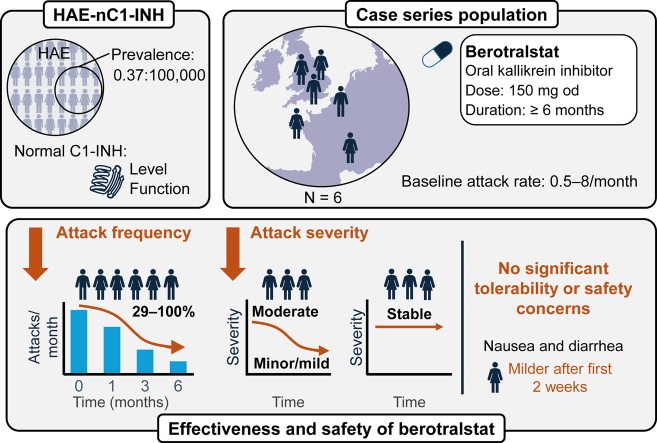

Hereditary angioedema (HAE) is a rare genetic disorder characterized by episodes of localized, spontaneous soft tissue swelling without urticaria.^1^^,^^2^ The swelling can affect multiple locations, including the face, upper respiratory tract, hands, feet, and gastrointestinal (GI) tract.^2^^,^^3^ Complications include pain, disability, and laryngeal edema leading to life-threatening airway compromise.^1^ HAE has negative effects on work attendance, quality of life, and mental well-being.^4^

Complement C1 inhibitor (C1-INH) is a member of the serpin (serine protease inhibitor) superfamily, functioning to regulate systems that result in the activation of bradykinin.^5^ HAE is classified by whether C1-INH is abnormal in both quantity and function (HAE-C1-INH type 1) or abnormal in function alone (HAE-C1-INH type 2).^2^ In a third group, C1-INH is normal in both quantity and function (HAE-nC1-INH type 3).^2^^,^^6^

The prevalence of HAE is difficult to ascertain but is estimated at 1.5 per 100,000 population.^7^^,^^8^ HAE-nC1-INH is less prevalent, with an estimated prevalence of 0.37 per 100,000 population in the United States.^9^ Swelling in HAE-nC1-INH affects the face and larynx more often compared with HAE-C1-INH.^10^^,^^11^

A number of genetic variants have been identified in patients with HAE-nC1-INH, but in some cases, no genetic cause has been found.^12^ Variants in the *FXII* gene have been most commonly described in the literature.^13^ Other pathogenic variants associated with HAE-nC1-INH have been identified in the *PLG, ANGPT1, KNG1, MYOF, HS3ST6, DAB2IP,* and *CPN1* genes.^14^ Among these, *F12* encodes a core protein of the kallikrein–kinin system (KKS), whereas the other genes affect kinin generation, kinin clearance, or Tie2/VEGF-mediated endothelial control.^14^

The pathophysiology of HAE-nC1-INH is not fully understood.^8^^,^^14^, ^15^, ^16^ However, two endotypes have been shown to be predominantly or exclusively bradykinin mediated.^2^^,^^14^^,^^16^ Uncontrolled kallikrein activity results in excess levels of a kinin ligand, which acts on the bradykinin B2 receptor to increase vascular permeability, causing soft tissue swelling.^17^ In some HAE-nC1-INH patients, laboratory analysis showed a dysregulation in the control of kallikrein, likely mediated by bradykinin.^18^ Additionally, C1-INH concentrate has been used to treat acute attacks in HAE-nC1-INH patients.^11^

The diagnosis of HAE-nC1-INH is complicated by the lack of an easily measurable specific biochemical marker.^16^^,^^19^^,^^20^ Diagnostic criteria include testing complement C4 levels and C1-INH level and function in patients with recurrent angioedema without urticaria.^21^ If C1-INH function and C1-INH and C4 levels are normal, genetic testing should be performed to identify known variants associated with HAE-nC1-INH and confirm the diagnosis of HAE-nC1-INH.^21^ When presenting with normal C1-INH level and function but in the absence of a known variant, clinical diagnosis is supported by a family history of angioedema and no response to antihistamines.^2^^,^^21^

Management of HAE includes treating acute attacks and preventing attacks in both the short and long term.^2^^,^^21^ Long-term prophylaxis (LTP) aims to reduce the frequency and duration of episodes, preventing life-threatening complications and improving patients’ quality of life.^21^

The 3 first-line options for LTP for HAE-C1-INH in the World Allergy Organization (WAO)/European Academy of Allergy and Clinical Immunology (EAACI) guidelines are berotralstat, lanadelumab, and plasma-derived C1-INH (pdC1-INH).^21^ Berotralstat is a once-daily oral plasma kallikrein inhibitor approved as LTP for HAE in the United States^22^ and Europe, including for HAE-nC1-INH.^23^ Both lanadelumab and berotralstat inhibit plasma kallikrein, binding at different sites: lanadelumab binds to the surface of the enzyme, whereas berotralstat binds deep within the active site.^24^ Berotralstat has been shown in a pivotal phase 3 randomized controlled trial to significantly reduce the frequency and duration of angioedema attacks when used as LTP in patients with type 1 and 2 HAE-C1-INH.^25^, ^26^, ^27^ However, no randomized controlled trials of berotralstat in patients with HAE-nC1-INH have been conducted. In practice, berotralstat has been provided to this patient group, and its efficacy has been reported in case studies.^28^^,^^29^ While treatment strategies targeting the KKS have demonstrated efficacy in HAE-C1-INH, their potential benefit in HAE-nC1-INH remains speculative and may be limited to specific disease endotypes.^8^^,^^11^^,^^30^

This retrospective case series analysis aimed to determine treatment-related outcomes of European HAE-nC1-INH patients treated with berotralstat and to describe relevant patient features, such as genotype and family history, in the context of diagnostic challenges. To our knowledge, this is the first multicenter retrospective case series on berotralstat therapy in the context of HAE-nC1-INH.

## Methods

This retrospective observational case series included data from patient records at 4 specialist centers in the United Kingdom (London) and France (Brest, Grenoble, and Lille).

Patients treated with berotralstat with a minimum of 6 months’ follow-up were included if clinicians diagnosed patients with HAE-nC1-INH by either a genetic assay demonstrating a known HAE-nC1-INH–associated variant (factor XII, plasminogen, kininogen, heparan sulfate-glucosamine 3-*O*-sulfotransferase 6, angiopoietin-1, or myoferlin); or if they had a reliable family history of angioedema, normal C1-INH level and function (performed in line with WAO/EAACI 2021 guidelines for HAE),^21^ and had no response to an appropriate course of antihistamine treatment.

Data were obtained retrospectively from patients’ medical records using a standardized template. Information was gathered regarding patient demographics, modalities of HAE diagnosis, clinical course of HAE before berotralstat, modalities of berotralstat therapy initiation, drug transitioning, treatment interruption, and berotralstat effectiveness, safety, tolerability, and discontinuation. Where available, patient-rated AngioEdema Control Test (AECT) scores indicating overall disease control were collected.^31^

To account for selection bias, the overall number of patients diagnosed with HAE-nC1-INH in the last 12 months at each center, as well as patients who initiated therapy with berotralstat but who had less than 6 months’ follow-up or discontinued therapy early, were recorded.

All patients consented to this research and publication of their data. Because data were obtained retrospectively from medical records, and because patient data were anonymized, institutional review board/ethics committee approval was not required. All procedures were performed in compliance with relevant regional laws.

## Results

### Center experience of patients with HAE-nC1-INH

At each center, 1 to 5 patients had been diagnosed with HAE-nC1-INH in the preceding 12 months. Each center had a caseload of between 7 and 15 patients with HAE-nC1-INH. In total, 9 patients with HAE-nC1-INH initiated berotralstat therapy; 6 are included in this analysis, 2 continued to receive therapy but did not have 6-month follow-up data, and 1 patient at the Grenoble center had discontinued berotralstat within 6 months of its introduction as a result of GI adverse effects. These adverse effects may have been amplified by the treatment’s not being administered with a meal as recommended.^23^ She stopped treatment on her own.

### Patient characteristics

The 6 patients were distributed across 4 centers. Two were diagnosed in childhood, 2 in adolescence, and 2 in adulthood (Table I). There were 4 female patients and 2 male patients, and all were White. The time from diagnosis to berotralstat initiation varied from 15 months to 7 years. All had normal C1-INH level and function. The majority had no variant identified by genetic testing; both the patients who did (patients 1 and 6) carried plasminogen gene variants. A family history of angioedema was reported by all patients who did not have a genetic variant identified. All had received antihistamine therapy for at least 6 weeks, and 4 of 6 patients had received therapy for more than 6 months without benefit. Patient 6 also had chronic spontaneous urticaria so had received 4-fold the maximum guideline-stated dose of antihistamine.^32^

**Table I:** Patient demographics

Patient no.	City, Country	Sex	Age at time of data collection / Life stage at diagnosis	Time (years) from diagnosis to initiation of berotralstat	Genetic variant	History of attacks and clinical presentation	Functional impact∗
1	Lille, France	M	76 years / Adulthood	6.75	Plasminogen	Attacks located at tongue and throat	Fatigue during attacks and anxiety impacting activities of daily living
2	London, United Kingdom	M	22 years / Adolescence	1.25	No	Periorbital swelling, tongue, feet, and hands with progressive onset of increasing severity and frequency over 2-month period	Affected ability to attend school with disfiguring facial swelling or to write with swollen hands
3	London, United Kingdom	F	21 years / Early childhood	5.5	No	Abdominal and throat swelling	85% school attendance only and problems with socialization, needed surgery (maxillectomy) and this was put on hold because of frequent facial and airway attacks
4	London, United Kingdom	F	51 years / Adolescence	7	No	Abdominal and facial swelling	Unable to work and difficulty in caring for daughter
5	Brest, France	F	44 years / Childhood (age 12)	5	No	Attacks of abdominal pain in childhood for 3 days; in adulthood, increased during 5 pregnancies and postpartum. Also experienced orofacial attacks in adulthood (3 of 22 crises in last 6 months).	Big impact on personal and professional life
6	Grenoble, France	F	75 years / Adulthood	7	Plasminogen	Tongue attacks	Retired; social life and some daily activities are subjectively affected

∗ Reported in health care professional’s words.

Before berotralstat initiation, all patients had active symptoms of HAE: all had recurrent attacks, all had orofacial attacks, and 2 reported regular acute hospital attendance. The 3 patients who had a recorded AECT scored <10 points, indicating poor disease control. The duration of continuous antihistamine therapy ranged between 12 weeks and several years. Four patients had been prescribed exogenous corticosteroids without benefit.

HAE had a significant effect on patients’ quality of life; 3 patients reported feelings of anxiety and 1 reported feelings of depression. One patient said that he was worried about going to university; another stated that it had a significant impact on both work and home life. Patient 2 described a “vicious cycle” between his attacks and anxiety.

### Transition to berotralstat

All patients had been previously prescribed other LTP. All patients had been prescribed tranexamic acid. Patient 6 was diagnosed with a stroke while receiving tranexamic acid therapy, which is a contraindication with this drug, and needed an alternative.^33^ Patient 1 also developed several thrombotic events while receiving tranexamic acid therapy and had tried lanadelumab, which did not control his attacks. Other patients changed therapy owing to lack of effectiveness of their current LTP and suboptimal control of their HAE.

The method of transition to berotralstat varied between patients (Table II). Two patients stopped tranexamic acid and immediately initiated berotralstat therapy. In one patient, the dose of tranexamic acid was concurrently increased while lanadelumab was stopped, and then berotralstat was initiated 15 days later. In another patient, the dose of tranexamic acid was weaned down a week after initiating berotralstat. Two patients had stopped their previous treatment several months before initiating berotralstat. No deviation from standard 150 mg daily dosing was reported during berotralstat therapy.

**Table II:** Treatment transition to berotralstat

Patient no.	Previous LTP for HAE	Description of transition	Berotralstat monotherapy after transition	AEs during transition
1	Tranexamic acid, lanadelumab	Lanadelumab stopped 15 days before berotralstat introduced. Tranexamic acid is increased (from 2 × 500 mg per week to 500 mg every other day)	No	None
2	Tranexamic acid 1 g 3 or 4 times a day	Tranexamic acid was weaned down 1 week after initiating berotralstat, reduced to 1 g per day gradually over 1 month, then stopped	No	None
3	Tranexamic acid 1 g 3 times a day	Tranexamic acid stopped immediately	Yes	None
4	Androgens, tranexamic acid	Tranexamic acid stopped immediately	Yes	None
5	Tranexamic acid, lanadelumab	Discontinuation of lanadelumab 4 months before berotralstat initiation	Yes	Nausea for first 2 weeks, then mild nausea and chronic diarrhea that did not require any symptomatic treatment
6	Tranexamic acid	Tranexamic acid withdrawn abruptly after ischemic stroke; initiation of berotralstat after resurgence of attacks 6 months later	Yes	None

### Safety

At the time of data collection, the duration of exposure to berotralstat ranged from 6 to 23 months. Five of 6 patients did not report any adverse events (AEs) related to berotralstat (Table II). Patient 5 experienced GI upset, in the form of nausea and diarrhea, at therapy initiation, which became milder after the first 2 weeks and did not lead to treatment discontinuation. No clinically significant changes were reported in routine blood tests (including full blood count as well as liver and renal function tests) monitored during transition in 4 patients. No patient receiving berotralstat therapy for 6 months or more discontinued for safety reasons. One patient (patient 1) discontinued treatment after 14 months owing to lack of effectiveness. This patient continued tranexamic acid and initiated pdC1-INH.

### Response to berotralstat

**Fig 1:**
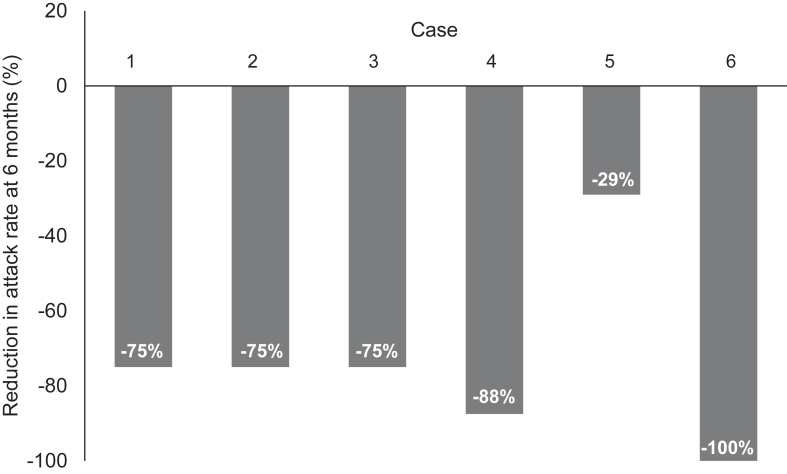
Percentage change in HAE attacks in patients with HAE-nC1-INH 6 months after transition to berotralstat. Percentage change calculated from number of attacks based on estimated mean number of attacks per month before initiation of berotralstat and at 6 months after initiation of berotralstat, with patient 1’s attack rate before therapy considered.

**Fig 2:**
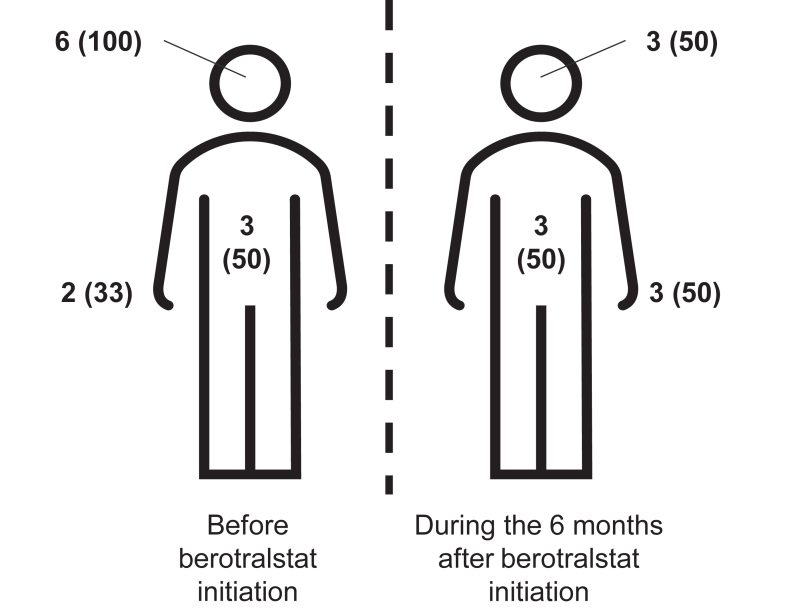
Overall distribution of attacks for all patients before and during 6 months’ berotralstat therapy, with numbers of patients (%) reporting attacks at corresponding anatomical site: orofacial (including laryngeal), abdominal, and peripheral.

Disease of 5 of 6 patients showed response to berotralstat within the first 3 months (Table III). In 3 of these patients, a further reduction in attack rates was noted between month 3 and month 6 after initiating berotralstat. Overall, within the first 6 months, a 29-100% reduction in attack rates was observed, based on individual case observations (Fig 1). However, in patient 1, the response observed was concomitant to a tranexamic acid dose increase; and his disease relapsed at the first attempt of tranexamic acid weaning. The number of subjective reports of orofacial attacks from the patients overall was reduced; one additional patient experienced attacks affecting the hands after taking berotralstat (Fig 2).

**Table III:** Berotralstat effectiveness

Patient no.	General impact of berotralstat (physician reported)	No. of attacks per month				Average duration of attacks	Average severity of attacks		Response to acute treatment (physician reported)
		Before transition	Month 1	Month 3	Month 6		Before transition	After transition	
1	Partially effective with decrease in AE frequency, but persistence of attacks reported; unclear if symptoms were due to berotralstat introduction vs tranexamic acid dose increase.	2∗	0.5	0.5	0.5	15 minutes†	Severe	Severe	Icatibant immediately effective (no repeat dosing needed)
2	Obvious rapid benefit. Attacks stopped almost completely without tranexamic acid. A few more hand swellings in first few weeks, but facial swellings stopped immediately.	4	4	2	1	Half a day	Moderate	Minor	NA—no significant attacks recently
3	Attacks now very occasional, mild, and self-limiting; able to attend school and take exams. After 1 year’s therapy, had orofacial surgery and had no associated angioedema attacks.	4	3	3	1	Quarter of a day	Moderate	Mild	No repeat dosing of icatibant required
4	Major impact—very few attacks after therapy for a few months since initiating berotralstat; any attacks are mostly self-limiting or respond to single 1.5 g dose of tranexamic acid.	8	4	2	1	Half a day	Moderate	Mild	Response in <4 hours
5	Reduction in frequency of HAE attacks.	3.5	1	1	2.5	<1 day	Severe	Severe	Good response to icatibant
6	NR	0.5	0	0‡	0	1 hour†	Severe	Severe	Icatibant very effective

*NA,* Not applicable; *NR,* not reported.

∗ Patient 1 attack rate before therapy.

† After administration of icatibant.

‡ Just 1 attack reported at month 4.

Attack severity was categorized as follows: mild, no impact on daily life; moderate, moderate impact on daily activities; and severe, laryngeal or abdominal attacks, or attacks with major impact on daily life. Severity of attacks was reduced in 3 patients from moderate to minor or mild. Three patients continued to experience attacks described as severe. Two of these patients received this severity grading on the basis of the above-shoulder location of their attacks.

No patients required HAE-related hospital treatment after initiation of berotralstat. The 5 patients who needed icatibant therapy to treat acute attacks after starting berotralstat showed a favorable response.

All 3 patients with AECT information showed an improvement in the score to 15, indicating well-controlled HAE. A notable impact on function and quality of life was described by these patients:

“Amazing, has given me a life back and forgotten there was ever a problem.”

“Massive positive impact and improvement in quality of life.”

“Massive improvement in quality of life, restored predictability, attacks happen but much less severe, frequent and it feels manageable.”

## Discussion

This case series describes 6 patients with HAE-nC1-INH who were treated with berotralstat for at least 6 months. Five of 6 patients showed improvement in the number of HAE attacks experienced per month after berotralstat initiation. However, in patient 1, the observed response was attributed to an increased dose of tranexamic acid. It is unknown why patient 5 noted an increase in attack frequency at 6 months compared with 3 months. Although there may be several reasons for this increase, one potential explanation for patients with disease that responds to medication initially and then exhibits less of a response could be a decrease in compliance, especially for daily oral therapies.^34^ We note that data on compliance were not collected in this case series. Additionally, the frequency of attacks in patients with HAE-nC1-INH is inherently variable, which may also contribute to fluctuations in response over time.^14^ There were no severe AEs noted. AEs reported in one patient were related to GI disturbance, consistent with findings of the pivotal APeX-2 study and the APeX-S open-label safety study in HAE types 1 and 2.^26^^,^^35^ It was recently suggested that a step-up dosing approach where patients begin with a dosage of 150 mg every third day could help reduce the incidence of these GI AEs, but this advice differs from the licensed dosage of berotralstat.^29^

The importance of effective LTP in HAE-nC1-INH patients is underlined by the fact that all included patients had experienced orofacial attacks before they began berotralstat therapy. These attacks, which may affect the larynx and upper airway, are potentially life-threatening and have been shown to be particularly common in patients with the plasminogen gene defect.^1^^,^^10^^,^^13^ Both patients with this gene defect experienced attacks affecting the tongue. Patient 6 experienced a reduction in attack frequency after beginning berotralstat therapy, but patient 1’s disease did not respond to berotralstat after tranexamic acid was weaned down, in line with expert consensus that suggests that tranexamic acid may be particularly effective in patients with this variant.^14^ This difference between the two patients is interesting in the context of emerging evidence that in some patients with plasminogen gene variants, kallikrein may be being bypassed.^36^ This hypothesis is strengthened by the fact that patient 1’s disease also did not respond to lanadelumab.

As previously identified, improved detection of HAE-nC1-INH is needed.^37^ The approach to diagnosis and treatment in this case series followed the principles set out in a recent consensus report from 31 global HAE-nC1-INH experts that aims to address this unmet need.^14^ The diagnostic challenges in HAE-nC1-INH are illustrated in the current case series in which a pragmatic real-world approach to inclusion criteria was chosen. Strict emphasis on the use of genotyping to diagnose HAE-nC1-INH would have excluded 4 cases from berotralstat treatment. Only 2 of 6 patients had identifiable genetic variants using the genotyping available in participating centers; however, 5 patients received durable benefit from this intervention. In a recent single-center case series, all 3 HAE-nC1-INH patients also had no relevant genetic variant but disease also responded to berotralstat, confirming these findings.^35^ The availability of genetic testing or physician familiarity with it may limit its use. Of physicians in the United States surveyed, only 43% tested patients with HAE-nC1-INH for *FXII* variants.^9^ Moreover, so far, some of the genetic variants identified in HAE-nC1-INH patients have been observed only in a single kindred.^38^

Patients whose disease responds to antihistamines are likely to have mast cell–mediated angioedema—an important differential diagnosis for HAE. Therefore, a trial of antihistamines is recommended in the WAO/EAACI guidelines for the diagnosis of HAE and was a criterion for inclusion of patients without demonstrated genetic defects in this case series. In a United States survey, 73% of physicians reported using antihistamine response to inform diagnosis in this patient group.^9^

In the current series, when defining the criteria for acceptance of a diagnosis of HAE-nC1-INH in the absence of genetic evidence, it was apparent that opinion varied between case contributors regarding how an adequate trial of antihistamine treatment should be defined, with national differences apparent. French case contributors would typically recommend that antihistamines should be prescribed for at least 3 months, whereas in the United Kingdom a shorter duration of 4 weeks was considered adequate. Elsewhere, the length of time during which 3 angioedema attacks might be expected has been proposed as a suitable trial of antihistamine receipt in this situation.^19^ The duration of a prophylactic trial is also influenced by the frequency of attacks, with authors more likely to accept a shorter duration if the attack frequency is very high. If disease of a patient with angioedema and no identified variant fails to show a response to 4-fold the maximum guideline-stated dose of an antihistamine, some clinicians would add in the anti-IgE monoclonal antibody omalizumab as the next step; this is included in a recent expert consensus.^14^^,^^39^ Others may consider targeting the KKS.

Another factor that may complicate the diagnosis of HAE-nC1-INH is the co-occurrence of more common histamine-mediated disorders.^29^ Patient 6 had a preexisting diagnosis of chronic spontaneous urticaria but had concurrent angioedema that did not respond to an anti-IgE monoclonal antibody in the same way the urticaria did. This demonstrates the importance of considering an alternative, potentially additional diagnosis if there is insufficient response to an appropriate treatment targeting the histaminergic pathway. Some centers reported routinely following a nonresponse to antihistamines or steroids by treating the next attack with icatibant to point to a diagnosis of HAE-nC1-INH before initiating LTP. Other real-world data demonstrate that similar practices are commonplace: 74% of physicians surveyed in the United States used response to a HAE-specific medication to inform diagnosis, with the majority prescribing icatibant.^9^

Future development of diagnostic tools may include better functional assays or clinical proteomics, measuring other markers of the KKS such as cleaved high-molecular-weight kininogen.^40^^,^^41^ Some authors have proposed that there may be a role for case finding in patients with disease that has not responded to antihistamines, even in the absence of a significant family history.^37^

In a previously reported case series, multiple prophylactic therapies were used to treat HAE-nC1-INH, including danazol, lanadelumab, pdC1-INH, and recombinant human C1-INH.^8^ Disease control improved in the majority of the 23 patients.^8^ A single case report of a patient with HAE-nC1-INH treated with berotralstat also showed efficacy, and the patient experienced no attacks during the first 6 months of therapy.^28^

More recently, a single-center case series of 3 patients who had a similar clinical profile to that described here showed similar effectiveness of berotralstat in reducing attack rates and in improving AECT scores.^29^

The findings of the current case series further support the value of identifying patients with HAE-nC1-INH because targeted options for LTP are now available, including berotralstat as the first licensed oral option. Robust estimates of the efficacy of LTP in HAE-nC1-INH are likely to remain elusive because of the challenges of recruiting sufficient numbers of patients with this rare endotype of HAE into placebo-controlled trials.^8^^,^^9^

This case series has limitations, including the difficulty of diagnosing patients with HAE-nC1-INH with absolute certainty in the absence of a demonstrated genetic defect. In addition, the lack of specific definition of an adequate duration of antihistamine receipt in non–genetically proven cases led to variation in practice, which could have led to inclusion of patients with histamine-mediated angioedema. Conversely, case contributors thought that a minimum of 6 weeks of antihistamine treatment would be adequate to demonstrate its lack of effectiveness, given the baseline attack frequency that had prompted them to prescribe LTP. It is therefore unlikely that any patients with histamine-mediated angioedema were included in this case series. Empirical evidence for this is provided by the observed reduction in attacks and improved disease control observed during kallikrein inhibition with berotralstat.

To account for case selection bias, we collected overall data for each center. This revealed only a single patient across all 4 centers in the 12 months before data collection who had initiated berotralstat therapy but discontinued it before 6 months. This demonstrates that there was no significant group of patients selected out owing to early discontinuation. The completeness and consistency of documentation, such as AECT scores, was affected by the fact that data were extracted retrospectively from routine clinical records.

All patients in this observational study were White. This should be considered in generalizing the findings to patients in other ethnic groups.

It is recognized that the retrospective nature of this study may have affected the quality of data collected, and that the sample size is too small to enable firm conclusions to be drawn on the effectiveness of berotralstat in all patients with HAE-nC1-INH. Because of the small sample size, no statistical comparisons were performed, precluding broader extrapolation of treatment effects.

While retrospective case collection has highlighted variability in some aspects of clinical practice across sites in 2 countries, the lack of a prospectively defined protocol for diagnosis and assessment may have reduced the comparability of results between centers and their generalizability to others.

In summary, this case series highlights the challenges encountered in the current diagnostic methods for HAE-nC1-INH. The results suggest that berotralstat is an effective option for LTP in patients with HAE-nC1-INH in the early European experience. Encouragingly, these results are in line with experience in type 1 and 2 HAE-C1-INH. No clinically meaningful tolerability or safety concerns regarding berotralstat were identified in this case series. This 4-center experience provides useful information to guide clinical decision-making in a population that currently has few, if any, evidence-based treatment options for LTP.

**Key messages**

• Berotralstat is a guideline-recommended, first-line, long-term prophylactic treatment for C1-INH–deficient HAE.
• Berotralstat showed effectiveness and a safety profile in line with previous experience in HAE endotypes 1 and 2.
• This real-world case series suggests patients with HAE with normal C1-INH may benefit from berotralstat as LTP.

## Disclosure statement

Medical writing assistance was provided by nspm ltd (Meggen, Switzerland), funded by 10.13039/100014935BioCryst Pharmaceuticals. BioCryst Pharmaceuticals selected authors on the basis of known early experience of use in the indication and designed the data collection form with their assistance.

Disclosure of potential conflict of interest: I. Boccon-Gibod declares receipt of payment or honoraria and support for attending meetings from BioCryst; and participation in advisory boards for CSL Behring, Takeda, and Kalvista. S. Sanges declares receipt of institutional grants or contracts from BioCryst; receipt of consulting fees from Novartis, Takeda, and Grifols; receipt of payment or honoraria from MSD; and receipt of institutional support for attending meetings from Shire, Sanofi-Genzyme, SOBI, Novartis, and BioCryst. M. Buckland has received previous grants, contracts, and/or consulting fees from CSL, Takeda, Pharming, and BioCryst Pharmaceuticals; has participated in data safety monitoring board or advisory board for Octapharma; and is chair of trustees for Immunodeficiency UK. The other author declares no relevant conflicts of interest.
