# Supplementary material for: Berotralstat effectiveness and safety in patients with hereditary angioedema with normal C1 inhibitor
Source: J Allergy Clin Immunol Glob. 2025 Sep 30;5(1):100575. doi: 10.1016/j.jacig.2025.100575 (PMC12573634; doi:10.1016/j.jacig.2025.100575)
Supplement: Supplementary Data [file mmc1.docx]

**Supplementary table 1: Patients’ family history of HAE**

| **Case** | **Affected family members** |
| --- | --- |
| 1 | None |
| 2 | Mother and maternal grandfather |
| 3 | Mother |
| 4 | Daughter |
| 5 | Sister, brother, grandmother |
| 6 | Daughter, mother and aunt (maternal), grandfather (maternal) |
